# Role of water-bridged interactions in metal ion coupled protein allostery

**DOI:** 10.1371/journal.pcbi.1010195

**Published:** 2022-06-02

**Authors:** Xingyue Guan, Cheng Tan, Wenfei Li, Wei Wang, D. Thirumalai

**Affiliations:** 1 Department of Physics, National Laboratory of Solid State Microstructure, Nanjing University, Nanjing, China; 2 Wenzhou Institute, University of Chinese Academy of Sciences, Wenzhou, Zhejiang, China; 3 Department of Chemistry, University of Texas, Texas, United States of America; University of Maryland School of Pharmacy, UNITED STATES

## Abstract

Allosteric communication between distant parts of proteins controls many cellular functions, in which metal ions are widely utilized as effectors to trigger the allosteric cascade. Due to the involvement of strong coordination interactions, the energy landscape dictating the metal ion binding is intrinsically rugged. How metal ions achieve fast binding by overcoming the landscape ruggedness and thereby efficiently mediate protein allostery is elusive. By performing molecular dynamics simulations for the Ca^2+^ binding mediated allostery of the calmodulin (CaM) domains, each containing two Ca^2+^ binding helix-loop-helix motifs (EF-hands), we revealed the key role of water-bridged interactions in Ca^2+^ binding and protein allostery. The bridging water molecules between Ca^2+^ and binding residue reduces the ruggedness of ligand exchange landscape by acting as a lubricant, facilitating the Ca^2+^ coupled protein allostery. Calcium-induced rotation of the helices in the EF-hands, with the hydrophobic core serving as the pivot, leads to exposure of hydrophobic sites for target binding. Intriguingly, despite being structurally similar, the response of the two symmetrically arranged EF-hands upon Ca^2+^ binding is asymmetric. Breakage of symmetry is needed for efficient allosteric communication between the EF-hands. The key roles that water molecules play in driving allosteric transitions are likely to be general in other metal ion mediated protein allostery.

## Author summary

Natural proteins often utilize allostery in executing a variety of functions. Metal ions are typical cofactors to trigger the allosteric cascade. In this work, using the Ca^2+^ sensor protein calmodulin as the model system, we revealed crucial roles of water-bridged interactions in the metal ion coupled protein allostery. The coordination of the Ca^2+^ to the binding site involves an intermediate in which the water molecule bridges the Ca^2+^ and the liganding residue. The bridging water reduces the free energy barrier height of ligand exchange, therefore facilitating the ligand exchange and allosteric coupling by acting as a lubricant. We also showed that the response of the two symmetrically arranged EF-hand motifs of CaM domains upon Ca^2+^ binding is asymmetric, which is directly attributed to the differing dehydration process of the Ca^2+^ ions and is needed for efficient allosteric communication.

## 1 Introduction

**Fig 1:**
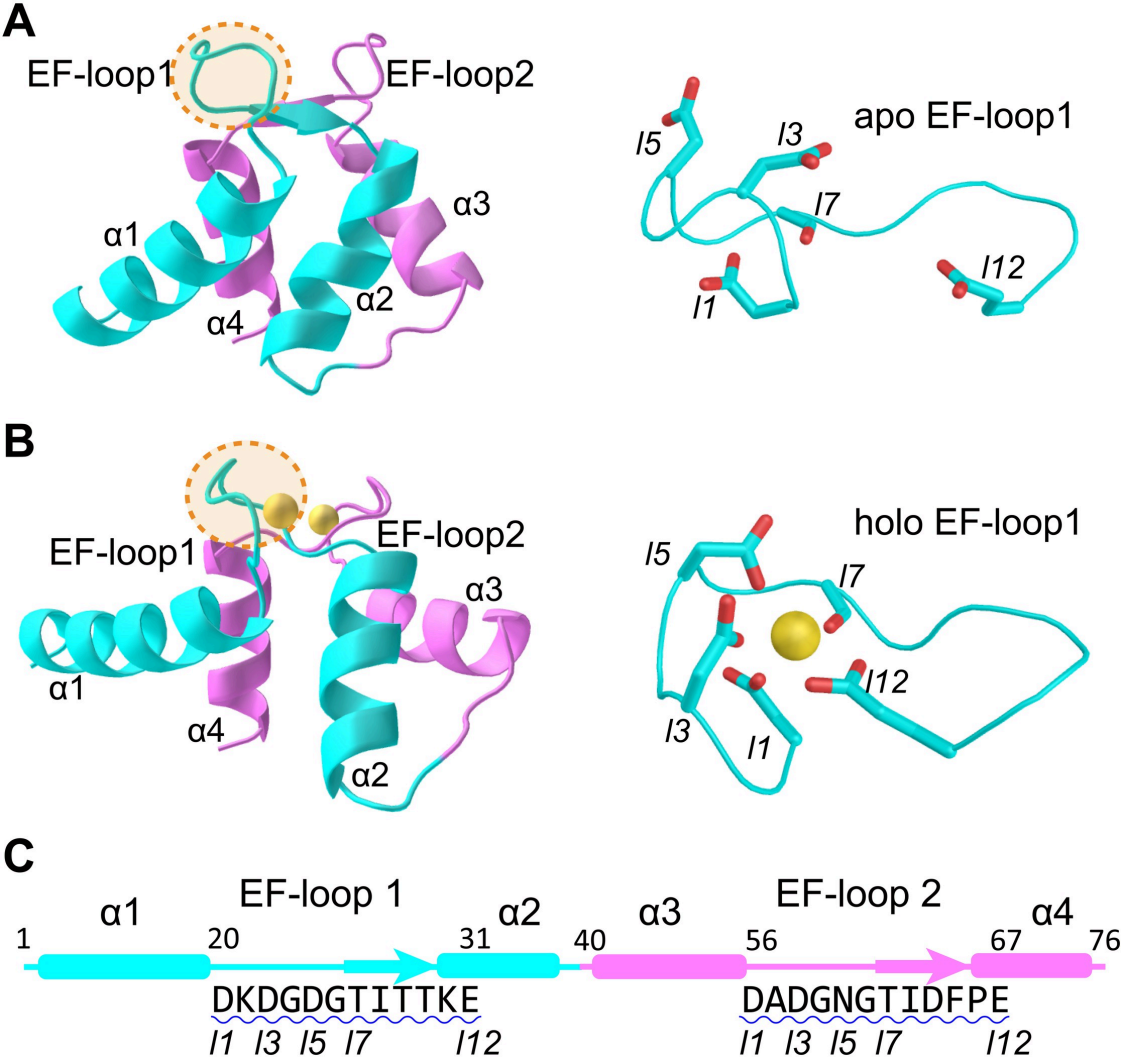
Structure and sequence of nCaM and the Ca^2+^ binding EF-loops. (A, B) Structure of nCaM in the apo state (A) and holo state (B). Helix-loop-helix motifs *α*1–EF-loop1–*α*2 (cyan) and *α*3–EF-loop2–*α*4 (violet) comprise two EF-hands in the N-terminal domain of Calmodulin. Yellow spheres correspond to Ca^2+^ ions. The details for one of the allosteric site in the loop region were also shown (right). (C) The sequence of nCaM and corresponding secondary structures. Ca^2+^ binding loops are underlined with blue wavy lines. The native ligands are marked with “*ln*” to indicate their position in the EF-loop.

Calmodulin (CaM), a versatile calcium (Ca^2+^) sensing protein expressed in all eukaryotic cells, is involved in a bewildering range of intracellular signaling processes [1]. Examples include activation of kinases [2, 3], muscle contraction [4], gene regulation [5], signal transduction [6–8], and apoptosis [9]. Binding of Ca^2+^, whose intracellular and extracellular concentrations differ by several orders of magnitude, to CaM results in a large conformational change, leading to the exposure of hydrophobic residues that serve as recognition sites for target proteins. CaM is composed of two nearly symmetric globular domains connected by a flexible central helix. Each domain consists of two helix-loop-helix motifs, termed EF-hands (Fig 1A and 1B), which are found in a large number of calcium-sensing proteins [10, 11]. The EF-hands chelate Ca^2+^, resulting in coordination to seven ligands arranged in a pentagonal bipyramid geometry (often involving one water molecule and five residues) with the negatively charged residues in the loop [11] (Fig 1B). In the apo state of CaM (Fig 1A), the helices in the EF-hand motif are arranged in an anti-parallel manner. Upon binding of Ca^2+^, the helices undergo substantial rearrangement into a nearly perpendicular conformation, exposing the hydrophobic sites which enable recognition and subsequent activation of target proteins [10, 12, 13]. Through such a mechanism, CaM initiates a variety of cellular processes.

In a more general context, Ca^2+^-triggered conformational changes in CaM is an example of allostery, which describes the structural changes that occur in enzymes and molecular machines at distances far from the site at which a ligand binds [14–16]. Nature utilizes such functional motions, which are triggered by binding of cofactors to specific sites in proteins and RNA, to execute a variety of functions [17–27]. Binding of cofactors at a certain site (allosteric site) induces structural rearrangements at a distant site (regulated site) in the enzyme, thereby modulating the downstream activity. By such allosteric (action at a distance) movements, small local changes are amplified over long distances, which allows for control and regulation of cellular signaling and functions [28]. Divalent metal ions, e.g., Ca^2+^ and Zn^2+^, have been widely utilized as effectors to trigger the allosteric cascade. However, due to the involvement of strong coordination interactions, the energy landscape dictating the metal ion binding and unbinding is intrinsically rugged. Despite the biological importance of metal ion coupled protein allostery, two key questions remain elusive, including: i) What strategy do metal ions utilize to overcome the ruggedness of the binding landscape and achieve rapid binding/unbinding? and ii) What physical interactions enable the propagations of the metal ion triggered allosteric signal to regulate the downstream processes? Because of the availability of rich experimental data and the typical allosteric features, CaM is an ideal system for answering these questions computationally [27, 29–32, 32–47].

Here, we used the bias-exchange metadynamics method [48] to investigate the Ca^2+^ binding coupled conformational changes in CaM domains by explicitly modeling Ca^2+^ in the simulations. We extracted the free energy landscape of the Ca^2+^ binding coupled conformational changes of CaM domains at atomic level and demonstrated the full picture of the allosteric motions of CaM [32, 49–51]. Our results revealed the key role of water-bridged interactions during Ca^2+^ binding and protein allostery. The bridging water molecules between Ca^2+^ and binding residues reduce the free energy barrier of ligand exchange landscape, and therefore the landscape ruggedness, by acting as a lubricant. This enables efficient Ca^2+^-ligand coordination and allosteric motions in the CaM. We propose that the water-bridged coordination is a general mechanism utilized in metal-coupled folding and allosteric communication in proteins. We also showed that Ca^2+^ binding leads to rotation of the EF-hand helices with the hydrophobic core as the pivot, a structural change that should precede recognition by target proteins for signal transduction. In addition, the molecular simulations revealed obvious asymmetry in the allosteric coupling of the symmetrically paired EF-hands: the EF-hand 1 (EF_1_) binds Ca^2+^ in a sequential manner by chelating to the N-terminal residues followed by coordination with the central residues, and finally to the C-terminal residues. In contrast, chelation of Ca^2+^ to the EF-hand 2 (EF_2_) is initiated either by interactions with the residues in the N- and/or C-terminal residues followed by coordination to the central residues. Similar results were obtained for the cCaM. Such a symmetry breaking process is likely involved in larger multi-domain complexes, such as the bacterial chaperonin, GroEL, in which ligand binding induces asymmetric response in different subunits.

## 2 Results and discussions

### Ca^2+^ binding is coupled to conformational changes of the EF-hand motifs

**Fig 2:**
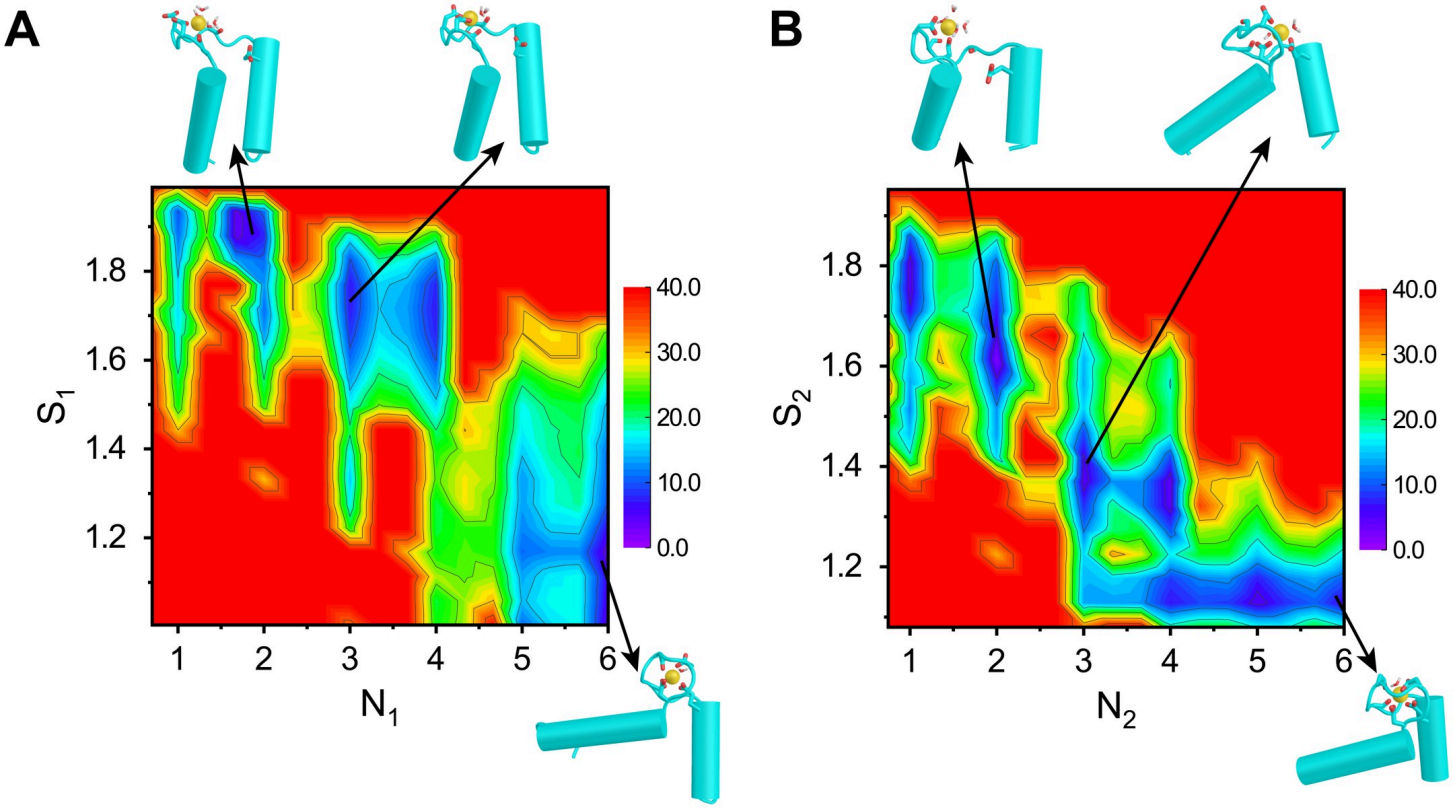
Free energy profiles of the Ca^2+^ coupled conformational transitions of EF_1_ (A) and EF_2_ (B) projected onto the collective variables (*N*_1_, *S*_1_) and (*N*_2_, *S*_2_), respectively. Representative conformations of the major basins in the landscape are also shown. The unit of the free energy is kJ/mol.

We use well-tempered bias-exchange metadynamics [48, 52] and the corresponding reweighting techniques [53] to extract the free energy landscape projected onto physically motivated multi-dimensional reaction coordinates characterizing Ca^2+^ binding and the conformational change. Quantitative analysis of the changes in these coordinates allows us to infer the mechanism of coupling between Ca^2+^ binding, the role of water, and the allosteric conformational changes in calmodulin. In particular, we use the “path collective variables” *S*_*α*_ (*α* = 1, 2, 3, and 4), defined in the **Methods and Materials** section, to describe the conformational changes of the two EF-hand motifs. Small and large values of *S*_*α*_ correspond to open and closed states, respectively. In order to assess the consequences of Ca^2+^ binding, we use the native coordination numbers *N*_*α*_ (Methods and materials), representing the number of native ligands (oxygen atoms of residues that are coordinated with Ca^2+^ in the native holo structure) that bind to Ca^2+^ during the allosteric transitions for the two EF-hand motifs, respectively. Fig 2 shows the free energy landscapes, *F*(*N*_1_, *S*_1_) and *F*(*N*_2_, *S*_2_), of the nCaM. For both EF_1_ and EF_2_, the conformational change of nCaM is tightly coupled to the extent of Ca^2+^ binding as indicated by the conformational distributions at different *N*_1_ and *N*_2_ values. When *S*_*α*_ (*α* = 1 or 2) is large and *N*_*α*_ (*α* = 1 or 2) is small, the closed state is more stable. Whereas when the native ligands are fully coordinated to Ca^2+^ the open structure is more stable (Fig 2 and Fig A in S1 Text). Interestingly, even with full coordination of native ligands (*N*_1_ = 6) to Ca^2+^, the EF_1_ samples a wide range of conformations as assessed by *S*_1_ fluctuations, suggesting the conformational plasticity of the open state. Conformational plasticity for the Ca^2+^ bound state, which was also observed in experiments, could be the major reason that the EF-hand motifs recognize and bind a variety of target proteins [54, 55].

Although the two EF-hand motifs have similar structures, comparison of Fig 2A and 2B shows that there are noticeable differences in the free energy landscapes upon Ca^2+^ binding. For example, when three or four ligands bind to Ca^2+^, the conformation of EF_2_ is poised to make a transition to the open-like structure, whereas EF_1_ remains in the closed-like structure. It is worth noting that the two EF-hand motifs were simulated as a whole in this work, and the conformational changes of the two EF-hands are not independent of each other. In the calculations of the free energy landscape of EF_2_, the EF_1_ is allowed to change its conformation. Therefore, the cooperativity between the two EF-hand motifs was included in the simulations. The free energy landscapes of the two EF-hand motifs shown in Fig 2 correspond to the projections of the overall free energy, but not the free energy landscapes of the isolated EF-hands. Detailed analysis shows that during the conformational transition, the two EF-hand motifs are tightly coupled. The open conformation of EF_2_ is stabilized when EF_1_ is in the open conformation with most of the associated native ligands bound to Ca^2+^. Thus, the behavior of EF_2_ is affected by the conformation of EF_1_ through long range allosteric interactions or action at a distance between the two EF-hands, which will be discussed further in later subsection. The observed differences in the allosteric communication are reasonable since the two EF-hand motifs have different amino-acid sequences in the Ca^2+^ binding loop. Consequently, the ensembles of structures with three or four native ligands bound to the Ca^2+^ should exhibit substantial differences in the two EF-motifs. Such an asymmetry in the Ca^2+^ binding and conformational transitions of the symmetrically arranged EF-hand pair in CaM domains likely plays an important role in signaling.

It is worth mentioning that conventional classic force fields often encounter difficulties in quantitatively reproducing the thermodynamic properties of the coordination bonds involving metal ions (especially Ca^2+^ or Mg^2+^) due to the lack of explicit consideration of charge transfer, polarization, and protonation/deprotonation effects [56–59]. For example, a recent study by Zhang and coworkers showed that the force field parameters derived from *ab initio* quantum calculations for the Ca^2+^ coordination in calmodulin depend on the loop conformations [56]. Meanwhile, a number of works have been devoted to improving existing force fields for better description of metal ion coordination [57, 58, 60, 61]. In an early work by Project and coworkers, a new set of Lennard-Jones (LJ) parameters for the calcium-oxygen pair were proposed to improve the quantitative description of the experimental Ca^2+^-formate affinity using the GROMOS96 and OPLS-AA force fields [57]. By refining the LJ parameters of calcium-oxygen pair for the OPLS-AA force field, Kahlen and coworkers [58] not only reproduced the experimental dissociation constant of calcium acetate, but also provided a better description of the key characteristics of the potential of mean force for the calcium acetate ion pair, including the relative probabilities of the contact ion pairs and the solvent-shared ion pairs. In this work, the OPLS-AA force field was used for the MD simulations of the Ca^2+^ coupled conformational change of calmodulin domains. According to the discussions in Ref. [57], the depths of the free energy minima involving the Ca^2+^ mediated interactions and the barrier separating them in the free energy landscapes of this work may depend on the details of the force field parameters. However, as will be discussed in the end of this section, the qualitative features of the free energy landscapes are insensitive to the details of the used force fields.

### Step-wise dehydration of Ca^2+^ and ligand coordination are cooperatively coupled

**Fig 3:**
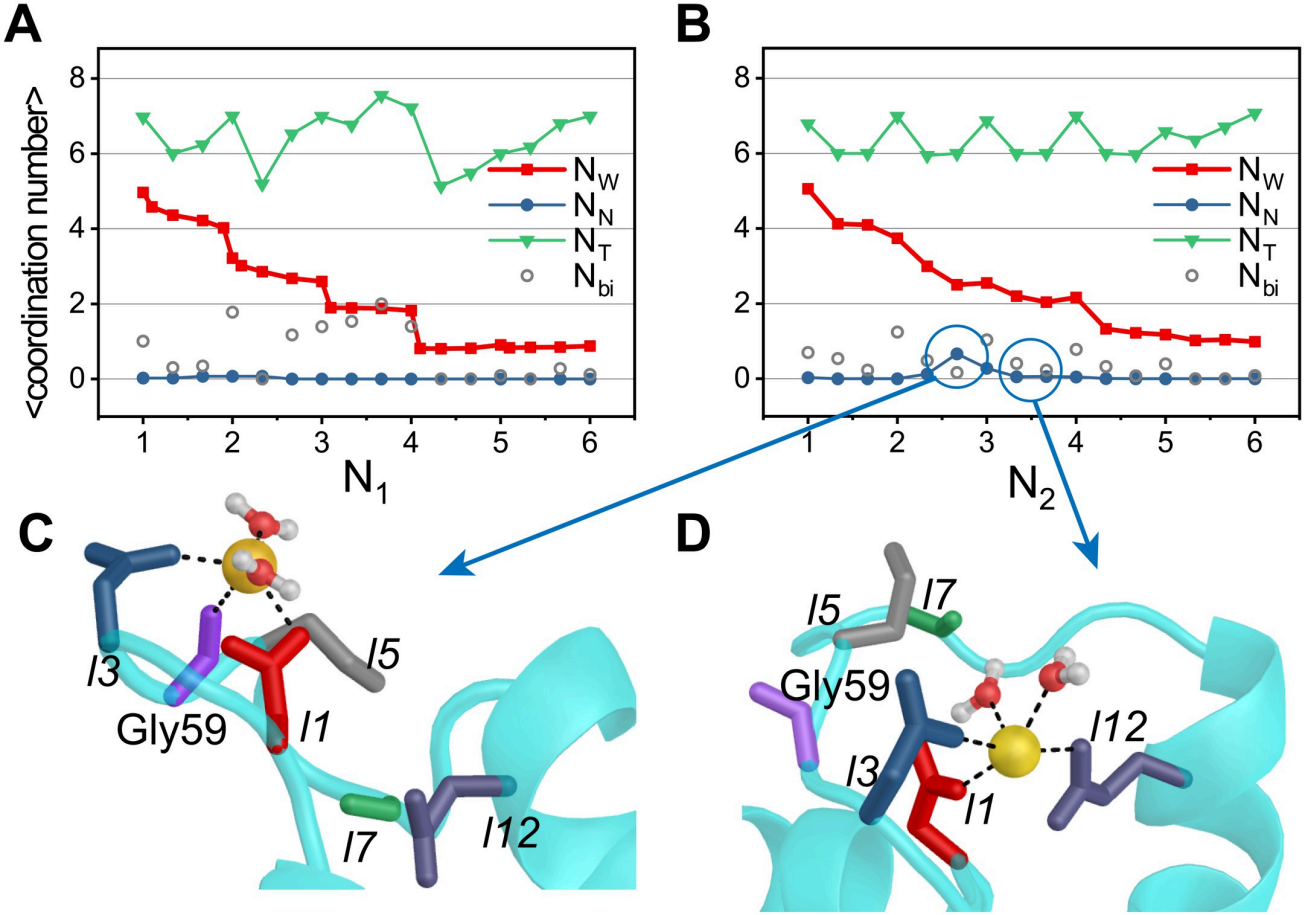
Number of coordinated water molecules (*N*_*W*_), non-native ligands (*N*_*N*_) as well as the total coordination number (*N*_*T*_) as functions of *N*_1_ and *N*_2_ for EF_1_ (A) and EF_2_ (B). Only one carboxyl oxygen atom of aspartate is coordinated to the Ca^2+^ in the native coordination structure. Nevertheless, the second carboxyl oxygen atom may also bind to the Ca^2+^ in the intermediate state of the coordination. The average number of such non-native bidentate ligands is also shown in panels A and B (N_*bi*_, gray). (C) The non-native ligand Gly59 (purple) coordinated to Ca^2+^ when *N*_2_ = 2 ∼ 3. (D) When *N*_2_ = 3, Glu67 (*l*12, purple) is coordinated to Ca^2+^, and the bond formed by Gly59 and Ca^2+^ is broken.

Our metadynamics simulations allow us to construct the changes in the coordination states of Ca^2+^, including the role that water and potential non-native ligands (those that are not present in the native open structure) play during the closed → open transition. Fig 3 shows the average of the total coordination number, *N*_*T*_ (sum of the number of water molecules and the number of ligands bound to Ca^2+^), as a function of *N*_*α*_ (*α* = 1, 2) for the nCaM. At a low value of *N*_1_ (Fig 3A), approximately five water molecules are coordinated to Ca^2+^. As *N*_1_ increases, water molecules are expelled, showing that dehydration occurs in steps. The number of water molecules expelled from Ca^2+^ and replaced by native ligands depends on *N*_1_. Upon binding of the first native ligand (*N*_1_ = 1), Ca^2+^ loses approximately two water molecules (Fig 3A). Notably, the first ligand is often the aspartate, which has two carboxyl oxygens. Although only one carboxyl oxygen atom is coordinated to the Ca^2+^ in the final native coordination structure, the second carboxyl oxygen atom may also bind to the Ca^2+^ in the early stage of the coordination (Fig 3A, gray). In comparison, one water molecule is ejected when *N*_1_ = 2, 3, and 4. With the binding of the last two native ligands, the number of coordinated water molecules remains unchanged. In the holo state (*N*_1_ = 6), only one water molecule is bound to the Ca^2+^, which makes the coordination number saturate at *N*_*T*_ = 7. (Fig 3A). During the coordination of native ligands, the pentagonal bipyramidal of the coordination center was roughly preserved (Fig B in S1 Text).

A similar process, with one crucial difference, occurs as Ca^2+^ coordination drives the conformational changes in EF_2_. During the binding of the native ligands (*N*_2_ ≤ 3 in Fig 3B), Ca^2+^ becomes transiently coordinated to non-native ligands, notably the backbone oxygen atom of Gly59. The carbonyl oxygen of Gly59 has a probability of ∼ 0.67 to come into the first ligand shell of Ca^2+^ during the binding of the third native ligand (see Table 1 and Fig 3C; the probability of the coordination is defined in S1 Text). As the binding process and conformational changes proceed further, the non-native ligands (oxygen atoms from Gly59) are replaced by the native ligands. Fig 3C and 3D explicitly show that non-native coordination of Gly59–Ca^2+^ is replaced by Glu67–Ca^2+^, a ligand present in the native holo state. Such mis-ligation and ligand-exchange may be necessary to minimize the free energy cost in the sharp dehydration of metal ion during the binding process [61–63].

**Table 1:** Probabilities of non-native ligand coordination and water-bridged native ligand coordination. O_*w*_ refers to oxygen in water. *N*_*α*_ is the number of coordinated native ligands.

EF_1_	*N*_1_ = 3	*N*_1_ = 4	*N*_1_ = 5
Ca^2+^–O_*w*_–Thr26	0.86	0.70	0.08
Ca^2+^–O_*w*_–Glu31	0.67	0.78	0.14
EF_2_	*N*_2_ = 3	*N*_2_ = 4	*N*_2_ = 5
Ca^2+^–O_*w*_–Asn60/Thr62	0.10	0.31	0.65
Ca^2+^–Gly59	0.67	0.05	0.02

### Asymmetry in the Ca^2+^ coordination pathways of the EF-hand pair

The results presented above strongly suggest that the conformational changes in the nCaM are coupled to the binding of Ca^2+^ with water playing a crucial role. Therefore, it is of interest to further clarify the order of binding of residues that are coordinated to Ca^2+^ in detail. Due to the nature of the metadynamics simulations, the kinetic information can only be indirectly gleaned using the present simulations. In particular, we can approximately extract the binding order based on the correlation analysis, as done in previous study [61].

**Fig 4:**
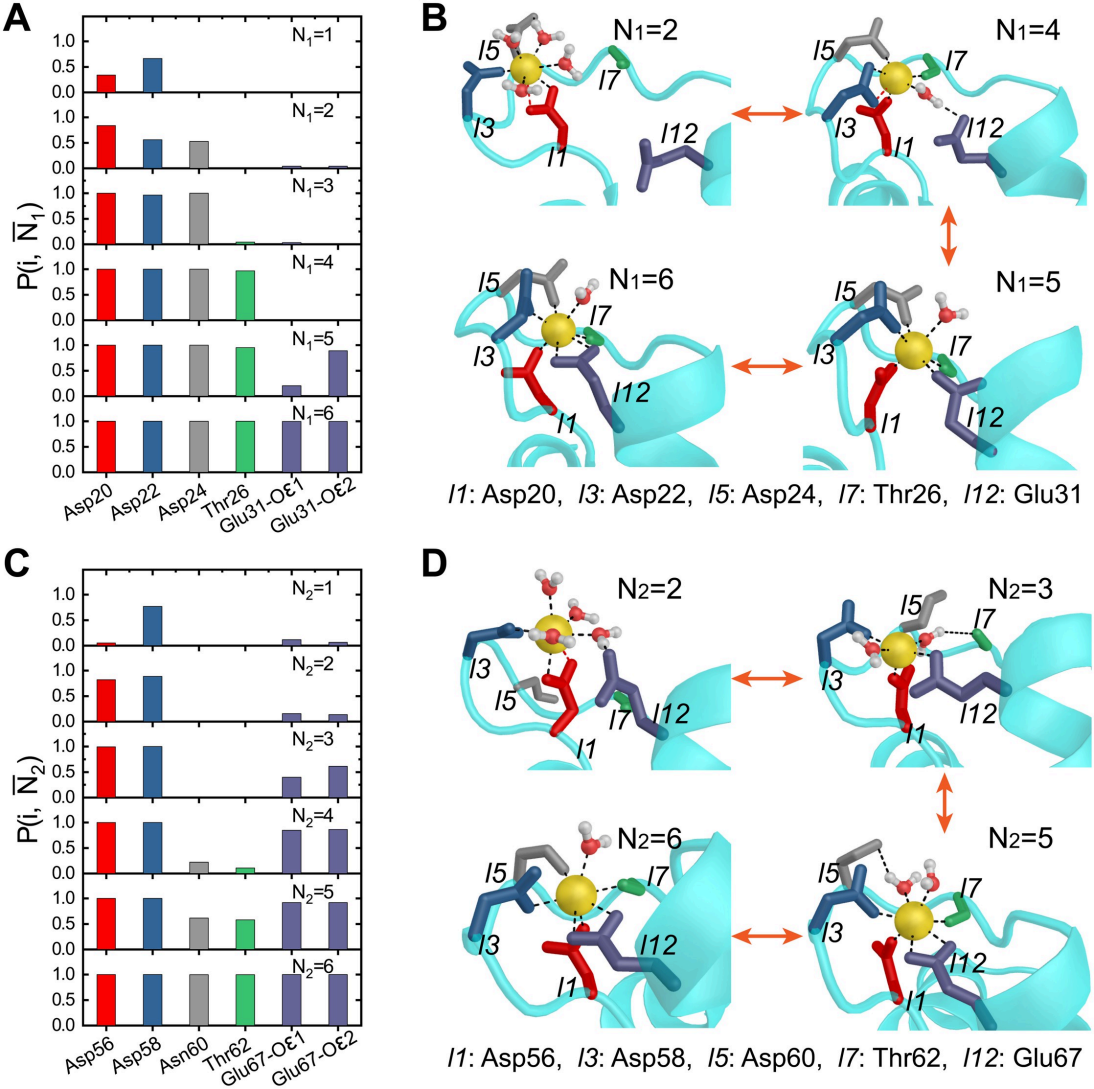
The Ca^2+^ binding sequence of EF_1_ and EF_2_. (A) Coordination probability $P(i,\bar{N_{\alpha }})$ for the six native amino acids residues in EF_1_. The residues coordinated to Ca^2+^ are shown in different colors. (B) Schematic representations of the binding process of the native ligands. (C) and (D) show the binding sequence of the EF_2_.

Fig 4A shows the coordination probability, $P(i,\bar{N_{\alpha }})$, (defined in S1 Text) for each ligand bound to the Ca^2+^ ion in the native state with the total native coordination number being one to six for the EF_1_ of nCaM. Using $P(i,\bar{N_{\alpha }})$ we can extract the binding order of the native ligands of EF_1_ to Ca^2+^. The representative structures are also shown (Fig 4B). Similar results for EF_2_ are shown in Fig 4C and 4D. For EF_1_, Ca^2+^ tends to bind first to the three negatively charged residues Asp20, Asp22, and Asp24 at the N-terminal part of the EF-loop. This observation is not surprising since negatively charged states of these residues contribute to the preferred binding and localization of Ca^2+^. With the progression of the Ca^2+^ chelation, several coordination bonds between the Ca^2+^ and water molecules need to be ruptured (Fig C in S1 Text), which may lead to the high free energy barrier between different coordination stages as shown in the two-dimensional free energy profiles (Fig 2).

Another possible reason for such a high free energy barrier is the dehydration of Ca^2+^ involving rupture of several coordination bonds between the Ca^2+^ and water molecules [64, 65]. The fourth native ligand, coordinated to Ca^2+^ in EF_1_, is the backbone oxygen of Thr26, which is at the central part of the EF-loop. Binding of Ca^2+^ to Thr26 results in the formation of a water-bridged interaction between Glu31 and Ca^2+^ (Fig 4B). In this structure, the oxygen of the water molecule is coordinated to Ca^2+^ as a ligand. At the same time, it forms a hydrogen bond with the side-chain oxygen from Glu31. We note that such a water-bridged coordination structure is quite similar to the intermediate structure identified in the crystal structure of a CaM mutant [47]. When the bridging water is finally expelled, the last two native ligands, side-chain oxygen atoms from Glu31, are coordinated to Ca^2+^. Overall, our results indicate a step wise binding of Ca^2+^ to EF_1_: first, Ca^2+^ is captured by an acidic residue (most probably Asp22); subsequently, the N-terminal part of the EF-loop plays a role in further coordination to Ca^2+^, establishing links between the incoming helix and the central *β* strands; finally, Thr26 and Glu31 are sequentially captured and bound to the Ca^2+^, leading to the native structure.

Interestingly, the results in Figs 2 and 4 show that once one of the two oxygen atoms from Glu31 are coordinated to Ca^2+^, the most stable conformation of EF_1_ switches to the open form, suggesting the crucial role of the highly conserved Glutamic acid at the 12^*th*^ position in the Ca^2+^ binding coupled conformational transition of nCaM [12, 47, 66]. A recent work based on nuclear magnetic resonance measurements also suggested the important role of the bidentate ligand Glu140 on the Ca^2+^ coupled conformational motions of the cCaM [46].

The molecular process of Ca^2+^ binding to EF_2_ is dramatically different (Fig 4C and 4D). Instead of binding to the native ligands by the sequence of N-terminal residues → central residues → C-terminal residues as in EF_1_, the Ca^2+^ binds initially to the N-terminal residues Asp56, Asp58 (or C-terminal residues Glu67), which is followed by the binding of the C-terminal residues Glu67 (or N-terminal residues Asp56, Asp58). Only at the final stage, it is bound to the central residues Asn60 and Thr62. Therefore, the Ca^2+^ binding to the EF_2_ follows the sequence N-terminal and/or C-terminal residues → central residues. The differing mechanism of coordination to Ca^2+^ probably arises from the divergence of their amino-acid sequences. For example, the “central” residues (especially at the 5^*th*^ position) in EF_1_ are more negatively charged compared to those in EF_2_. Consequently, coordination of these residues to Ca^2+^ occurs at a relatively early stage.

We also found that before Ca^2+^ binds to the central residues Asn60 and Thr62, a non-native ligand Gly59 is coordinated with Ca^2+^ (Fig 3B, 3C and 3D). Consequently, the later steps of the Ca^2+^ binding to EF_2_ must involve ligand exchange between the non-native ligands and the native ligands. Interestingly, during the ligand exchange, water molecules bridge the Ca^2+^ and the native ligands. As we show below, water mediated coordination reduces the free energy barrier of the formation of native coordination bonds. For EF_2_, the water molecules were found during the formation of coordination of Ca^2+^–Asn60 and Ca^2+^–Thr62, which is consistent with the observation that there is a tendency to capture an extra water molecule as a ligand leading to a decreased contribution for the backbone oxygen of Thr62 [50]. In experiments [47], Asp64 was mutated to Asn64 for the EF_2_. Interestingly, it was shown that the mutated EF_2_ has similar binding mechanism as EF_1_, namely, the N-terminal part of the loop are coordinated to Ca^2+^ earlier than the C-terminal ligands [47]. These results suggest that the charged state of the residues to which Ca^2+^ is coordinated plays a crucial role in the mechanism of Ca^2+^ binding, and hence allostery [28].

The cCaM exhibits a similar Ca^2+^ binding order except that the order of events in the EF_3_ and EF_4_ in the cCaM corresponds to those of the EF_2_ and EF_1_ in the nCaM (Fig D in S1 Text), as expected from the similarity of the sequences of the respective Ca^2+^ loops. As shown in Fig 1 and Fig D in S1 Text, the residue at the *l*5 position of the EF_1_ (EF_4_) in the nCaM (cCaM) is charged, whereas those in the EF_2_ (EF_3_) in the nCaM (cCaM) are neutral. The stepwise dehydration of the Ca^2+^ in the cCaM was also observed (Fig E in S1 Text). We performed simulations for the nCaM and cCaM separately. Therefore, we cannot characterize the coupling between the two domains. Previous studies showed that binding of the full-length calmodulin to target proteins may produce positive cooperativity between the two domains for Ca^2+^ binding [67]. Simulations have also shown that application of a pulling force may introduce cooperativity for the allosteric motions of the two domains [27].

### Ca^2+^ binding-induced rotation of EF-hand helices

**Fig 5:**
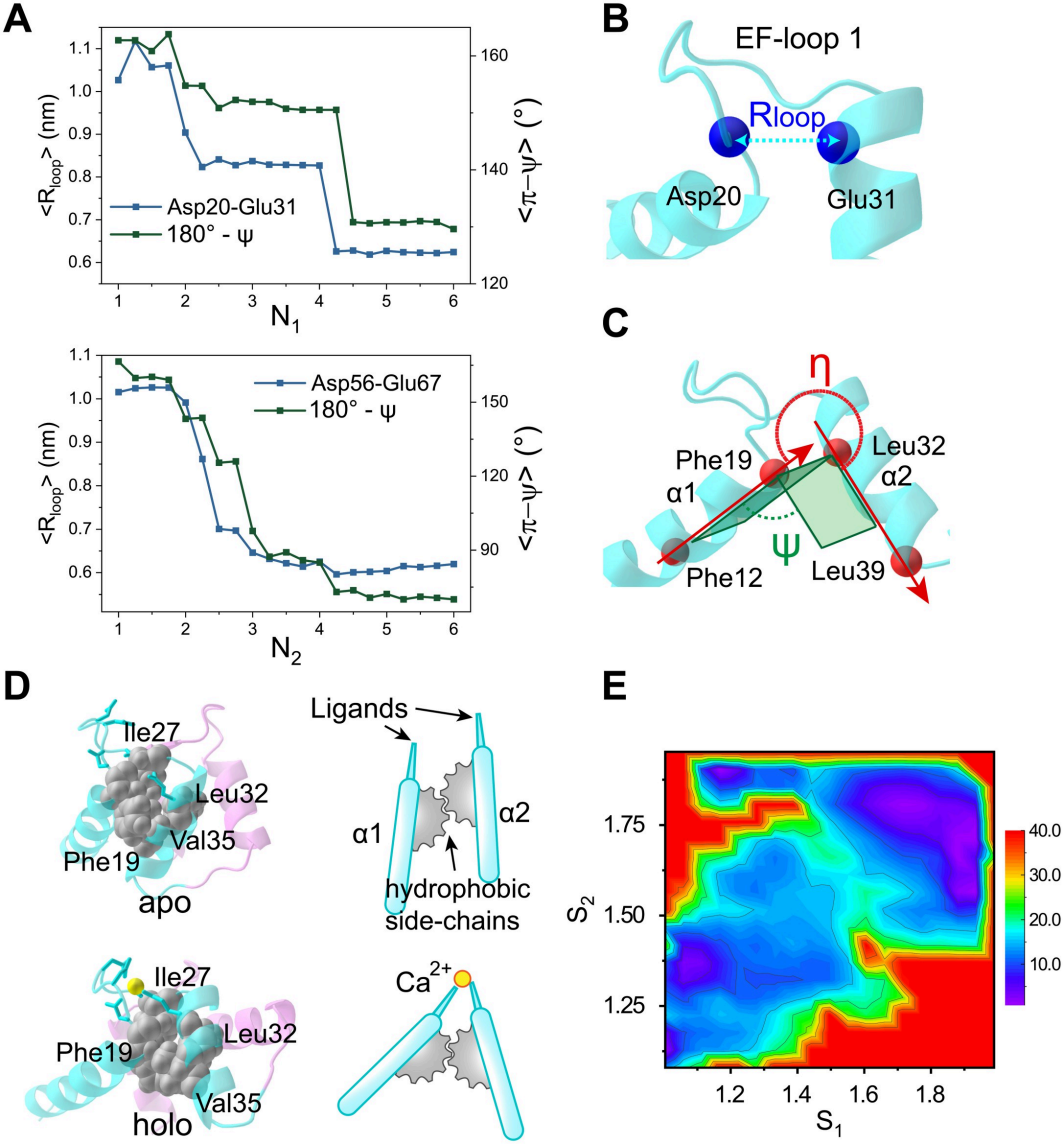
Ca^2+^ binding induced rotation of EF-hand helices. (A) The distance between close ends of EF-loop 1 (represented by the C_*α*_ atoms from Asp20 and Glu31) and the dihedral angle characterizing the rotation of the two helices of EF_1_ as a function of *N*_1_ (upper panel). The same results for the EF_2_ are shown in the lower panel of (A). (B) Schematic diagrams illustrating the end-end distance of EF-loop1 and (C) the inter-helical angle and diheral angle of EF_1_. The end-end distance of EF-loop is represented by the distance between C_*α*_ atoms: EF-loop1 by Asp20–Glu31, and EF-loop2 by Asp56–Glu67. The direction of helices are represented by vectors pointing from one residue’s C_*α*_ atom to another residue’s C_*α*_ atom: *α*1 by Phe12–Phe19, *α*2 by Glu31–Leu39, *α*3 by Leu48–Val55, and *α*4 by Phe68–Lys75. The dihedral angles Ψ are defined by the C_*α*_ atoms of the residues Phe12–Phe19–Glu31–Leu39 for EF_1_ and Leu48–Val55–Phe68–Lys75 for EF_2_. (D) Schematic diagram of the Ca^2+^ binding induced EF-hand helix rotation. Black spheres indicate the hydrophobic core in EF_1_. (E) Free energy profile of nCaM conformational transition projected onto *S*_1_ and *S*_2_. The free energy scale is in kJ/mol.

Structures of nCaM show that there are several hydrophobic residues located around Ile27 and Ile63 whose non-polar side-chains stack against each other to form the center of the hydrophobic core [54, 68] (Fig 5). During the Ca^2+^ binding and conformational transition, the hydrophobic cluster formed by these residues, including Phe16, Phe19, Ile27, Leu32, Val35, Ile52, Val55, Ile63 and Phe68, are almost rigid (Fig F in S1 Text). Comparison of the structures in the apo- and holo- states suggests that the two helices of the EF-hand rotate around the hydrophobic cluster during the conformational transition.

To demonstrate the coupling between the Ca^2+^ binding and the EF-hand opening, we calculated the distance between the EF-loop ends (*R*_*loop*_, distance between the C_*α*_ atoms of the first and twelfth residues, see Fig 5B) and the dihedral angle for the rotation of the two helices (Ψ, defined by the C_*α*_ atoms of four residues selected from the *α*-helices, as shown in Fig 5C) of the two EF-hands, respectively. The results in Fig 5A show that *R*_*loop*_ decreases upon Ca^2+^ binding, thus pulling the ends of the EF-hand helices close together (blue lines). In particular, coordination of the fourth (third) ligand to Ca^2+^, which corresponds to the binding of the terminal residues of the EF-hand loops to the Ca^2+^ by water bridged interactions (direct interactions) in EF_1_ (EF_2_), has the most prominent effect in changing the distance between the two close ends. Meanwhile, the dihedral angles formed by the two helices increase (red lines), indicating the rotation of the EF-hand helices. In the process of rotation, the hydrophobic cluster, which is stable during the Ca^2+^ binding, acts as the pivot (Fig 5D). We also defined the angle *η* to characterize the in plane rotation of the two helices, and similar results can be observed (Fig G in S1 Text).

The results described above show that the large conformational change in the two EF-hand motif occurs at different stages of the coordination (Fig 2). We simulated the two EF-hand motifs of the individual CaM domain as a whole system, which implies that cooperative interactions between the two EF-hand motifs are automatically included. The differences in the free energy profiles for the two EF-hand motifs is related to the high cooperativity. Analysis of the correlation between the coordination processes of the two EF-hand motifs demonstrates that the Ca^2+^ binding in the EF_1_ proceeds before interaction with EF_2_ (Fig H in S1 Text). When N_1_ is less than 5, N_2_ predominantly takes on the values of 1 or 2, and both the EF_1_ and EF_2_ mainly stay in the closed conformation (Fig 2). As shown in Fig 5E, the conformational changes in the two EF-hands mostly follow the diagonal line in the two-dimensional free energy landscape, which suggests tight coupling and cooperativity between the two EF-hand motifs. With the full coordination of the EF_1_ (N_1_ = 5 and 6), it switches to the open conformation. Due to the tight coupling between the two EF-hand motifs, Ca^2+^ coordination induced stabilization of the open conformation of the EF_1_ tends to promote the closed to open switching of the EF_2_ conformation, even though the coordination of the EF_2_ is not fully completed. Consequently, we observe that the major conformational switching occurs at N_1_ = 5 for the EF_1_, but it occurs at N_2_ = 3 for the EF_2_ as shown in the free energy profiles (Fig 2). The tight coupling between the two EF-hands is consistent with previous experimental observations [29, 41].

Taken together, these results demonstrate that the rotation of the EF-hand helices is coupled to Ca^2+^ binding, which is consistent with the free energy landscapes shown in Fig 2. Ca^2+^ binding induced rotation of the EF-hand helices was also noted in a simulation study [69].

### Bridging water molecules lower the free energy barrier between the allosteric states

As mentioned above, water-bridged interactions play a crucial role during Ca^2+^ binding, and hence in the allosteric transitions in CaM. To further demonstrate the role of water molecules, we performed detailed analyses of the ensemble of conformations containing water-bridged coordinated structures. We assume that a water-bridged coordination is formed if the following conditions are satisfied: (i) the distance from oxygen of water molecule (*O*_*w*_) to Ca^2+^ is less than 2.8Å; and (ii) the hydrogen bond is formed between water and the native ligand. For each residue listed in Table 1, the Ca^2+^-water-native ligand conformation exists at certain stages of the binding process with high probability. These results suggest that before the final chelation process, the native ligands already develop indirect interactions with Ca^2+^ mediated by water molecules. For example, after binding of Ca^2+^ to five of the native ligands of EF_2_, the last native ligand, Asn60 or Thr62, is not “free”, but has a probability of 0.65 to be bridged to the Ca^2+^ by a water molecule (Table 1).

**Fig 6:**
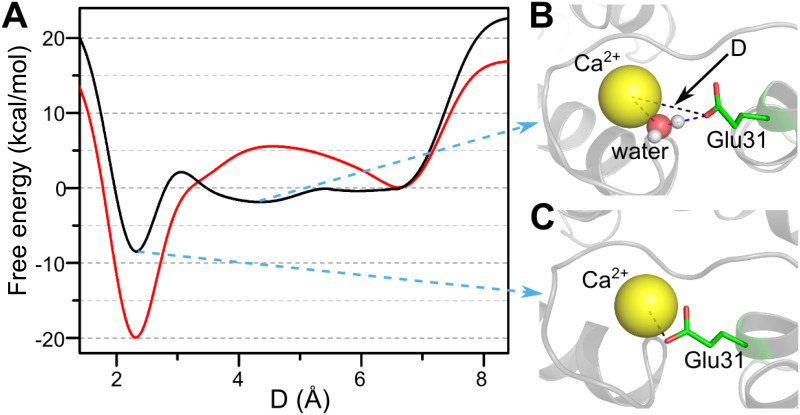
Free energy profiles of water bridged coordination of Glu31 side-chain oxygen atoms to the Ca^2+^. (A) Potential of mean force for the binding/unbinding process of Glu31 to Ca^2+^ as a function of the minimum distance between side-chain oxygen atoms of Glu31 and Ca^2+^. The black line illustrates the results with the water molecules being realistically treated. The red line shows the results with electrostatic interactions involving the bridging water molecules being turned off. (B) The water-bridged coordination of Glu31 oxygen and Ca^2+^, where *D* = 4.3Å is a local free energy minimum. (C) shows the direct coordination bond of Glu31 oxygen and the Ca^2+^, which corresponds to the most stable state at *D* = 2.3Å.

To quantitatively evaluate the contribution of water molecules to the Ca^2+^ binding process, we characterize the binding involving water bridge in Glu31 to the Ca^2+^ in the final step of the Ca^2+^ coordination to EF_1_. We used well-tempered metadynamics to extract the free energy profile along the collective variable *D*, which is defined as the distance between Ca^2+^ and the closest side-chain oxygen of Glu31 (Methods and materials). As a control, we also designed a system in which the electrostatic interactions between the bridging water molecule and Glu31 side-chain atoms were turned off. The differences between the two simulations reflect the net effect of the bridging water on the binding free energy landscape. When the interaction of the bridging water includes the electrostatic potential, an intermediate is present in the free energy profile at *D* ≈ 4.3Å (see Fig 6A, black line), in which the Glu31 is bridged to the Ca^2+^ by the bridging water molecule (Fig 6B), leading to a low free energy barrier for ion coordination. In contrast, when the electrostatic interactions involving the bridging water molecule are switched off, a high free energy barrier is observed (∼ 6kJ/mol, shown in Fig 6A, red line). We surmise that water reduces the free energy barrier by bridging the Ca^2+^–Glu31 interaction, therefore speeding up the coordination between the Ca^2+^ and Glu31.

Both energetic and entropic effects of the bridging water contribute to the free energy barrier reduction. The bridging water, which is coordinated to the Ca^2+^, forms a hydrogen bond with the surrounding ligand (e.g., the side chain oxygen atoms of Glu31 in Fig 6B), introducing an intermediate structure during the ligand exchange. The water mediated hydrogen bond interaction stabilizes the intermediate structure, thus reducing the free energy barrier for ligand exchange. This is an energetic effect. On the other hand, due to the water bridged interactions, the accessible volume in the conformational space of the surrounding ligand is limited, which reduces the entropy barrier for the formation of the coordination bond with Ca^2+^. It is difficult to quantify the magnitudes of the two effects separately based on the current simulations because their values will doubtless depend on the force field, although the qualitative results would be similar. We propose that the contribution of water molecule to the binding of metal ions should be a common feature in other metal-ion induced biological processes.

As shown in early studies, conventional classic force fields with fixed partial charges may encounter difficulties in providing quantitative description of the thermodynamic properties of Ca^2+^ mediated coordination interactions [56–58]. To test the sensitivity of the major results of this work to the used force field, we performed simulations with the modified OPLS-AA force field for the Ca^2+^ coordination using the parameter given elsewhere [58] and calculated the one-dimensional free energy landscapes. The results showed that the qualitative features of the free energy landscapes are insensitive to the details of the used force fields, although the modified OPLS-AA force field gives a less stability of the native coordination bond (Fig I in S1 Text). Even with the modified force field, we observe significant contribution of the bridging water in reducing the ruggedness of the ligand exchange free energy landscape compared to control simulations without water bridged interactions. This finding is consistent with the results shown in Fig 6.

## 3 Conclusions

Allosteric transitions are ubiquitous in biology [15]. Nowhere is it more prominent in modulating large conformational changes over long distances (nearly 20Å) than it is in CaM induced by Ca^2+^ binding. Using all-atom molecular dynamics simulations in conjunction with metadynamics, which enables efficient sampling of the conformational space, we have established that in the Ca^2+^ binding coupled allosteric transition process water-bridged interactions play crucial role. In addition to participating in the native coordination of Ca^2+^ as widely observed in the crystal structures of metalloproteins [70], the transient bridging water molecules between Ca^2+^ and binding residues during the Ca^2+^ binding/unbinding process tend to reduce the ruggedness of ligand exchange landscape by acting as a lubricant, facilitating the Ca^2+^ coupled protein allosteric motions. The high free energy cost dehydration of Ca^2+^ occurs in steps with the number of waters lost at different stages being compensated by gain in chelation to the charged residues in the loops of the EF-hands. The overall picture that emerges is that there is a coordinated interplay between ligand binding, loss of water around the cation, and subsequent changes in the conformations of the protein. We propose that similar mechanisms, with water playing an important role, might also mediate allosteric transitions in molecular machines, such as molecular chaperones, molecular motors, and cell adhesion molecules, in which allosteric transitions are often triggered by cations. The ability to undergo dehydration depends on ion charge density, which for Ca^2+^ is in the optimal range [71]. Our results also demonstrated that there is an asymmetry in the Ca^2+^ binding and conformational transitions in the EF-hand pair of isolated CaM domains despite the overall structural similarity. The Ca^2+^-coupled closed to open transitions in the two EF-hand motifs occur by an entirely different mechanism, which could facilitate the differing functional requirements of CaM.

A key event in the Ca^2+^ coupled allostery of CaM is the dehydration of Ca^2+^. Had the charge density been even somewhat larger, as is the case in Mg^2+^ the dehydration would not occur rapidly enough to induce the functionally required allosteric transition. Thus, the ease of fast dehydration in Ca^2+^ may well have been a consequence of evolution in CaM and other Ca^2+^ systems, such as the lever arm in myosin motors.

## 4 Methods and materials

### Molecular dynamics simulations

We used the NMR structure of *Xenopus* nCaM for the holo form with 1J7O [68] as the Protein Data Bank (PDB) entry. The apo form with the same sequence (residue 1 to 76) was taken from the NMR structure of *X. laevis* CaM with PDB code 1CFD [54]. All MD simulations were carried out using GROMACS 2019.6 [72] with the OPLS/AA force field [73]. The Ca^2+^-nCaM system was solvated in the SPC/E water box together with Na^+^ and Cl^−^ ions to mimic the ion concentration of 0.15*M*. The whole system had 15,117 atoms, of which 13,929 atoms were from water. We used the periodic boundary condition where the size of unit box is ∼ 49.2 × 55.9 × 56.7 Å^3^. Energy minimization was performed before the MD simulations. Before running metadynamics simulations the Ca^2+^-nCaM systems were heated to 300K in the NVT ensemble and then equilibrated for 10 ns MD under NPT conditions, with *P* = 1atm and *T* = 300K. Periodic conditions were used and electrostatic interactions were calculated using the particle-mesh Ewald algorithm. The simulations for the cCaM were conduced similarly.

### Collective Variables (CVs) in metadynamics

We used the “bias-exchange” [48] form and “well-tempered” [52] algorithm of metadynamics to accelerate conformational sampling, using the PLUMED 2.6.1 package [74]. The CVs used to study the Ca^2+^-binding coupled conformational change of CaM were chosen to describe the rate-limiting events involving the binding of Ca^2+^ and the subsequent conformational transition of the protein.

The events driving the closed to open transition involve movement of a number of particles from distant parts of CaM domains. In order to capture such an allosteric movement, we need suitable reaction coordinates. Since the major events in the closed → open transition are triggered by Ca^2+^ binding, we constructed CV involving the oxygen atoms (ligands) of the residues in the holo structure that are coordinated to Ca^2+^. In terms of these native ligands, we define the collective variables, *N*_*α*_ (*α* = 1 and 2 (3 and 4) for EF_1_ and EF_2_ (EF_3_ and EF_4_), respectively, of the nCaM and cCaM) to describe the number of native ligands that are coordinated to Ca^2+^:

$$\begin{array}{c} N_{\alpha }=\sum_{i\in \text{native}\;\text{ligands}}k_{i}^{\alpha }, \end{array}$$ (1)

where $k_{i}^{\alpha }$ is defined using the switching function,

$$\begin{array}{c} k_{i}^{\alpha }=\{\begin{array}{cc} 1 & \text{for}\;r_{i}^{\alpha }\leq d_{0}\\ \frac{1-{\left(\frac{r_{i}^{\alpha }-d_{0}}{r_{0}^{\alpha }}\right)}^{4}}{1-{\left(\frac{r_{i}^{\alpha }-d_{0}}{r_{0}^{\alpha }}\right)}^{6}} & \text{for}\;r_{i}^{\alpha }>d_{0} \end{array} \end{array}$$ (2)

In Eq (2), $r_{i}^{\alpha }$ is the distance from the *i*^*th*^ native ligand to the *α*^*th*^ Ca^2+^. By native ligand we mean the oxygen atoms from distinct amino acids that are linked to Ca^2+^ and are present in the native (NMR) holo structure. We chose *d*_0_ = 2.5Å and *r*_0_ = 0.02Å. For all the Asp residues, the two oxygens from the same residue were considered “degenerate”, namely, when more than one oxygen is coordinated to Ca^2+^, we consider only one of them to be a native ligand. However, for the Glu residues in the 12^*th*^ position of the EF-loops of nCaM and cCaM, two oxygens are treated as individual ligands. We treated coordination of Ca^2+^ to Asp differently from binding to Glu because in the holo structure of the nCaM and cCaM (PDB entry 1J7O and 1J7P, respectively) Ca^2+^ is coordinated to both oxygen atoms of Glu but only to one of the Asp oxygen atoms.

We also used the “path collective variables” calculated using,

$$\begin{array}{c} S_{\alpha }=\frac{\sum_{i=1}^{2}ie^{-\lambda d(X_{i}^{\alpha },X\left(t\right))}}{\sum_{i=1}^{2}e^{-\lambda d(X_{i}^{\alpha },X\left(t\right))}}, \end{array}$$ (3)

where *α* = 1, 2, 3, and 4 dictates the *α*^*th*^ EF-hand, and $d(X_{i}^{\alpha },X\left(t\right))$ is the mean square deviation from the closed (*i* = 1) and open (*i* = 2) structure for a given conformation *X*(*t*). A small value of *S*_*α*_ corresponds to closed structure and a large value of *S*_*α*_ corresponds to open structure. We used λ = 20 in our simulations.

To use the bias-exchange method with metadynamics, we designed 5 replicas. In the first replica, there is no bias in the CVs. In each of the other four replicas, a bias was applied to one of CVs, including *N*_1_, *N*_2_, *S*_1_, and *S*_2_ for the nCaM simulations, and *N*_3_, *N*_4_, *S*_3_, and *S*_4_ for the cCaM simulations. Exchange between replicas was attempted every 10,000 MD steps, and each replica lasts for 800ns. In the well-tempered algorithm, we used a bias factor of 200 for the rescaling of the Gaussian height, and 0.3kJ/mol as the initial Gaussian height. More detailed descriptions of the simulation procedure can be found in S1 Text and Figs J, K and L in S1 Text.

### Water-bridged coordination of Glu31 to the Ca^2+^

We also used well-tempered metadynamics to study water-bridged coordination of Glu31 to the Ca^2+^ for the nCaM. The minimum distance between the Ca^2+^ and two oxygen atoms of Glu31 is chosen as the collective variable *D* [74], which we define as:

$$\begin{array}{c} D=\frac{\sigma }{\text{ln}\;\sum_{i}\;\text{exp}(\sigma /\|r_{i}\left\|\right)}, \end{array}$$ (4)

where *r*_*i*_ (*i* = 1, 2) is the distance between each oxygen and the Ca^2+^, and *σ* = 5000Å. The well-tempered rescaling factor is 12, and the initial Gaussian height is 0.2kJ/mol.

In the control simulation, once a water molecule comes into the first ligand shell of Ca^2+^ and simultaneously within 3.5Å distance of any of the two oxygen atoms of Glu31, the electrostatic interactions between the water and the side-chain heavy atoms of Glu31 are turned off. This was accomplished by modifying the source code of GROMACS package.

To achieve better convergence we use a restraining potential at *D* = 6.5Å to limit the region of the phase space accessible during the simulation:

$$\begin{array}{c} V_{wall}\left(D\right)=K{\left(D-D_{wall}\right)}^{2},\;\text{for}\;D>6.5Å, \end{array}$$ (5)

where *K* = 50*kJ*/*mol*/Å^2^ and *D*_*wall*_ = 6.5Å. We performed 20 independent 100ns simulations for each system, and then calculated the ensemble average to obtain the free energy reported in Fig 6. Comparison of the simulations with and without electrostatic interactions allows us to assess the role water plays in modulating the binding of Ca^2+^.

The numerical data used in all figures are included in S1 Data.

## Supporting information

S1 Text Text for the details of simulation methods and calculations of coordination probability, and supplementary figures showing the one-dimensional free energy profiles, coordination geometry analysis, coordination probability of the C-terminal domain of calmodulin, convergence test, free energy landscapes with a modified OPLS-AA force field, and some additional results. (PDF)

S1 Data Numerical data underlying relevant figures. (XLSX)
