# Supplementary material for: Role of water-bridged interactions in metal ion coupled protein allostery
Source: PLoS Comput Biol. 2022 Jun 2;18(6):e1010195. doi: 10.1371/journal.pcbi.1010195 (PMC9197054; doi:10.1371/journal.pcbi.1010195)
Supplement: S1 Text — (PDF) [file pcbi.1010195.s001.pdf]

Supplementary information

Role of Water-bridged interactions in Metal Ion Coupled Protein Allostery

Xingyue Guan[1,2,*], Cheng Tan[1,*], Wenfei Li[1,2,†], Wei Wang[1,†], and D. Thirumalai[3,†]

[1]Department of Physics, National Laboratory of Solid State Microstructure, Nanjing University, Nanjing 210093, China

[2]Wenzhou Institute, University of Chinese Academy of Sciences, Wenzhou, Zhejiang 325000, China

[3]Department of Chemistry, University of Texas, Austin, TX, 78712 USA

[*]These authors contributed equally to this work

[†]wfli@nju.edu.cn, wangwei@nju.edu.cn or dave.thirumalai@gmail.com

Supplementary Methods

Well-tempered Metadynamics algorithm

The metadynamics algorithm was used to enhance conformational sampling in simulations so that Free Energy Surfaces (FES) could be reliably computed. In complex systems, it is necessary to identify physically reasonable collective variables (CVs) to describe large scale conformational transitions. In the canonical metadynamics algorithm, the system is biased through the addition of a sum of repulsive Gaussian terms to the system potential energy function. The biasing potentials are usually applied to the CVs. The total biasing potential applied on a set of CV, $Y(x) = \big(Y_1(x), Y_2(x), \ldots, Y_n(x)\big)$, at time t during the simulation can be expressed as [1]:

$$V\big(Y(x), t\big) = \sum_{t'=0,\delta t,2\delta t,\ldots}^{t'<t} h \prod_{i=1}^{n} \exp\left(-\frac{\big(Y_i(x) - y_i(t')\big)^2}{2\delta_{y_i}^2}\right), \tag{S1}$$

where h is the height of each Gaussian potential, δ_{y_i} is the Gaussian width associated with the CV $Y_i(x)$, δt determines the time step at which the biasing potentials are added. In order to accelerate the convergence of metadynamics, the well-tempered algorithm was designed [2], in which the height of the Gaussian potentials is systematically attenuated during the course of the simulations using $e^{-V_{meta}(Y(x),t)/\Delta T}$, where ΔT represents a characteristic energy [2]. The form of the biasing potential used in our simulations is

$$V_{meta}\big(Y(x), t\big) = \sum_{t'=0,\delta t,2\delta t,\ldots}^{t'<t} h_0 e^{-V_{meta}\big(Y(x(t')),t'\big)/\Delta T} \prod_{i=1}^{n} \exp\left(-\frac{\big(Y_i(x) - y_i(t')\big)^2}{2\delta_{y_i}^2}\right). \tag{S2}$$

where h_0 is the initial Gaussian height. In practice, the rate of decrease is controlled by the bias factor $\eta = (T + \Delta T)/T$ [3], where T is the system temperature. Combining the replica exchange methodology and metadynamics, the bias-exchange metadynamics was designed to enhance the sampling efficiency[4]. As an inherent feature of the algorithm, the converged metadynamics runs enable the calculation of the canonical probability distribution of the CVs directly. However, the statistics of other degrees of freedom is affected by the bias, and require "reweighting" techniques to reconstruct the unbiased canonical probability distribution[5]. In particular, for well-tempered metadynamics, the reconstruction algorithm is described in Ref.[6], and can be found in the PLUMED package [7], which is used in our work.

Technical details of the simulations

During the metadynamics simulations, we applied restraining potentials to the values of the MSD (mean square deviation) of a conformation with respect to the folded states to limit the accessible region of phase space sampled by the protein. The use of such restraining potentials physically prevents unreasonable unfolding of nCaM. The functional form of the restraining potential is [3]:

$$V_{wall}\left(d(X_i^\alpha, X(t))\right) = \kappa(d(X_i^\alpha, X(t)) - d_0^\alpha)^2, \quad \text{for } d(X_i^\alpha, X(t)) > d_0^\alpha \tag{S3}$$

where the energy constant $\kappa = 0.8\text{kJ/Å}^4$, $d(X_i^\alpha, X(t))$ is the MSD from the closed ($i = 1$) or open ($i = 2$) structure of the αth ($\alpha = 1, 2$) EF-hand for a given conformation $X(t)$, the limit d_0^α ($\alpha = 1, 2$) is chosen as $d_0^1 = 41\text{Å}^2$ for EF_1 and $d_0^2 = 36\text{Å}^2$ for EF_2. The terminal residues with high flexibility were not included in the calculations of the MSD values. For better convergence, we prevent Ca^{2+} ions from leaving too far from the binding sites by applying a lower limit to the coordination number, as one of the reaction coordinates.

Convergence of metadynamics simulations

We sampled the solution conformations of nCaM and the associated Ca^{2+} binding using well-tempered bias-exchange metadynamics with 5 replicas (in four of which biasing potentials were applied). The simulations were initiated from random conformations, and were continued for 800ns. We assessed the convergence of the metadynamics simulations by ensuring that the free-energy differences between all the major populated regions for each CV reach a steady state. For the four biased replicas, this condition was satisfied after about 400ns of sampling (see Fig J in S1 Text). In addition, as an embedded property of well-tempered metadynamics, the automatically rescaled Gaussian height guarantees convergence, and avoids fluctuations around the correct free energy value [2, 8]. Therefore, we checked the height of the biasing Gaussian potentials during the simulations. As can be seen in Fig K in S1 Text, 200ns after sampling starts, all the Gaussians are less than 1/10 of the initial height, which also indicates the convergence of our metadynamics simulations. In addition, we analyzed the evolution of two dimensional FESs during the simulations. As an example, FESs of N_1-S_1 at different simulation times are shown in Fig L in S1 Text. The evolution of FESs also show that they are converged after 400ns of simulations. Thus, we conclude that the calculated FESs as a function of CVs are well converged

in our simulations.

Coordination probability

Binding pathways are quantified using the probability, $P(i, \overline{N_\alpha})$, that the ith residue is coordinated to Ca^{2+} when the total ligand number of EF-hand α is $\overline{N_\alpha}$ ($\overline{N_\alpha} = 1, 2, \ldots, 6$). We define $P(i, \overline{N_\alpha})$ using

$$P(i, \overline{N_\alpha}) = \frac{\int k_i^\alpha P(k_i^\alpha, N_\alpha) \theta_{\overline{N_\alpha}}(N_\alpha) \, \mathrm{d}k_i^\alpha \, \mathrm{d}N_\alpha}{\int P(k_i^\alpha, N_\alpha) \theta_{\overline{N_\alpha}}(N_\alpha) \, \mathrm{d}k_i^\alpha \, \mathrm{d}N_\alpha}, \tag{S4}$$

where N_α and k_i^α are defined in equation (1) and equation (2) (in the main text), respectively, and $\theta_{\overline{N_\alpha}}(N_\alpha)$ is a step function:

$$\theta_{\overline{N_\alpha}}(N_\alpha) = \begin{cases} 1 & \text{for } N_\alpha \in [\overline{N_\alpha} - 0.5, \overline{N_\alpha} + 0.5) \\ 0 & \text{for } N_\alpha \notin [\overline{N_\alpha} - 0.5, \overline{N_\alpha} + 0.5) \end{cases} \tag{S5}$$

$P(k_i^\alpha, N_\alpha)$ is a two dimensional probability distribution of the variables k_i^α and N_α, which cannot be directly calculated as the average over time from the metadynamics. We use the reweighting techniques described in Ref.[6] to reconstruct the unbiased distribution. We calculated the two-dimensional probability distributions on a 60×60 grid. Then we constructed bins on the N_α dimension as $(0, 0.5), [0.5, 1.5), [1.5, 2.5), \ldots, [4.5, 5.5), [5.5, 6]$, and binned any $N_\alpha \in [\overline{N_\alpha} - 0.5, \overline{N_\alpha} + 0.5)$ into integer values $\overline{N_\alpha}$.

The occurrence probability of the water-bridged coordination has the same form as equation (S4), except for the definition of k_i. For the water-bridged coordination, $k_i = 1$ when the oxygen of water is within 2.8Å of the Ca^{2+} and at the same time within 3.5Å of the oxygens from a native residue involved in ligand formation, and is zero otherwise.

References

[1] Laio, A.; Parrinello, M. Escaping free-energy minima. *Proc. Natl. Acad. Sci. U.S.A.* **2002**, *99*, 12562–12566.

[2] Barducci, A.; Bussi, G.; Parrinello, M. Well-Tempered Metadynamics: A Smoothly Converging and Tunable Free-Energy Method. *Phys. Rev. Lett.* **2008**, *100*, 020603.

[3] Tribello, G. A.; Bonomi, M.; Branduardi, D.; Camilloni, C.; Bussi, G. PLUMED 2: New feathers for an old bird. *Comput Phys Commun* **2014**, *185*, 604–613.

[4] Piana, S.; Laio, A. A bias-exchange approach to protein folding. *J. Phys. Chem. B* **2007**, *111*, 4553–4559.

[5] Marinelli, F.; Pietrucci, F.; Laio, A.; Piana, S. A kinetic model of trp-cage folding from multiple biased molecular dynamics simulations. *PLoS Comput. Biol.* **2009**, *5*, e1000452.

[6] Bonomi, M.; Barducci, A.; Parrinello, M. Reconstructing the equilibrium Boltzmann distribution from well-tempered metadynamics. *J Comput Chem* **2009**, *30*, 1615–1621.

[7] Bonomi, M.; Branduardi, D.; Bussi, G.; Camilloni, C.; Provasi, D.; Raiteri, P.; Donadio, D.; Marinelli, F.; Pietrucci, F.; Broglia, R. A.; Parrinello, M. PLUMED: A portable plugin for free-energy calculations with molecular dynamics. *Comput. Phys. Commun.* **2009**, *180*, 1961–1972.

[8] Bonomi, M.; Parrinello, M. Enhanced Sampling in the Well-Tempered Ensemble. *Phys. Rev. Lett.* **2010**, *104*, 190601.

[9] Kahlen, J.; Salimi, L.; Sulpizi, M.; Peter, C.; Donadio, D. Interaction of charged amino-acid side chains with ions: An optimization strategy for classical force fields. *J. Phys. Chem. B* **2014**, *118*, 3960–3972.

Supplementary Figures

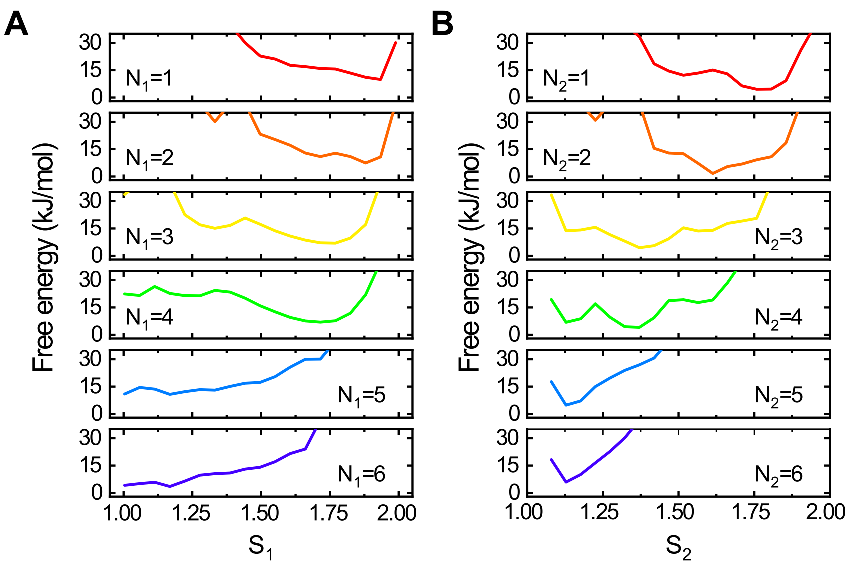

Fig A: One-dimensional free energy profiles of the Ca^{2+} coupled conformational transitions of EF_1 (A) and EF_2 (B) along the collective variables S_α (α=1 and 2 for the EF_1 and EF_2, respectively) at different binding stages described by N_α, which is the number of native ligands that are coordinated to Ca^{2+}.

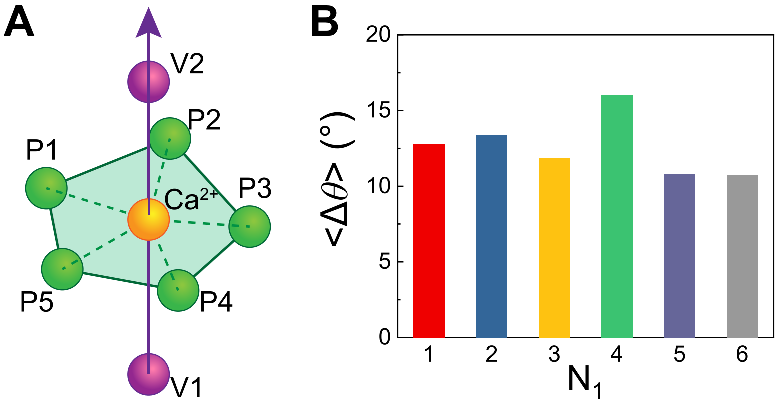

Fig B: Analysis of Ca^{2+} coordination geometry. (A)Cartoon diagram illustrating the ideal pentagonal bi-pyramid geometry of Ca^{2+} coordination. Pi ($i = 1 - 5$), V1, and V2 represent the ligands of the Ca^{2+}. In the ideal pentagonal bi-pyramid geometry, the angles (θ^0_{Pi}) formed by Pi-Ca^{2+}-$V2$ ($i = 1 - 5$) are 90° and the angle (θ^0_V) formed by $V1$-Ca^{2+}-$V2$ is 180°. (B) Average deviation of the angles defining the pentagonal bi-pyramid geometry of Ca^{2+} coordination at different stage of the Ca^{2+} coordination for the nCaM. The deviation of the coordination angle is given by $\Delta\theta = |\theta - \theta^0|$, with θ and θ^0 being the angles calculated for the snapshots sampled by MD simulations and the corresponding angles for the ideal pentagonal bi-pyramid geometry, respectively. The average was performed among the six coordination angles and all the sampled structures at different coordination stage. The $< \Delta\theta >$ value is $\sim 7.7^o$ for the native coordination structure of the nCaM based on the crystal structure. One can see that the average deviation of the coordination angles at different stage of the coordination in the MD simulations varies between 10.8° and 16.0°, suggesting that the pentagonal bi-pyramid geometry of the Ca^{2+} coordination is roughly preserved.

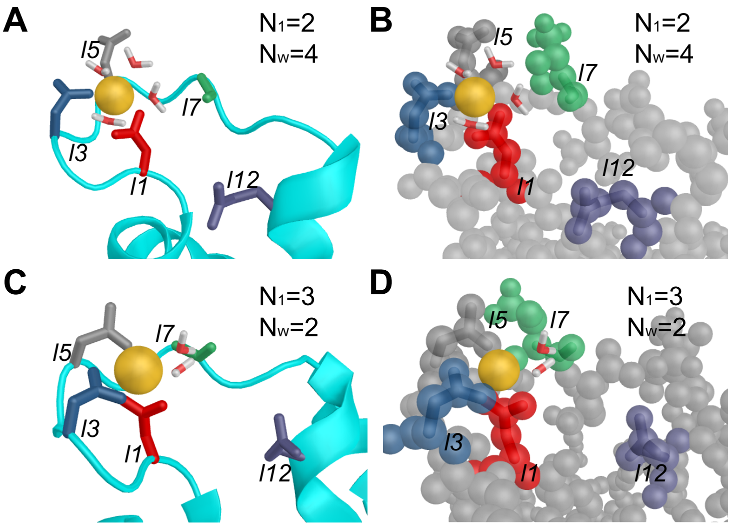

Fig C: Dehydration of Ca^{2+} is coupled to the binding of native ligands from $N_1 = 2$ to $N_1 = 3$ in EF_1. (A) and (B) show the coordination conformation of Ca^{2+} when $N_1 = 2$, using "new cartoon" (A) and "sphere" (B) drawing methods. $N_w = 4$ represents that four water molecules are coordinated to Ca^{2+}. (C) and (D) show the coordination of Ca^{2+} when $N_1 = 3$ and $N_w = 2$. These figures illustrate the decrease of N_w by two when the third native ligand is bound to Ca^{2+}. Possible reasons are the potential coordination of two side-chain oxygens from one aspartate to Ca^{2+}, such as Asp20 ($l1$) in (C), and volume exclusion effects at the binding site in protein, as shown in (D).

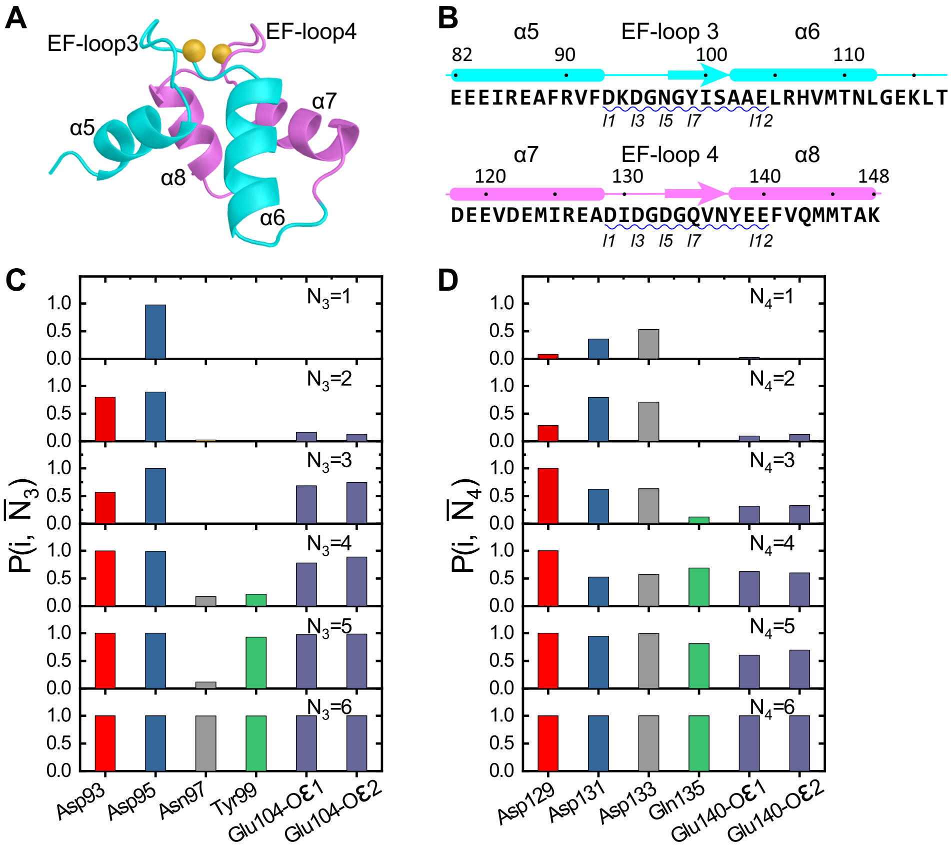

Fig D: Ca^{2+} binding coupled to conformational changes of the EF-hand motifs in cCaM and molecular mechanism of the Ca^{2+} binding. (A)Cartoon representation of the three-dimensional structure of the cCaM in the holo state. (B) Sequence of cCaM and corresponding secondary structures. (C) Coordination probability $P(i, \overline{N_\alpha})$ for the six native liganding residues of EF_3. Different liganding residues are colored in different colors. (D) Same as C but for EF_4.

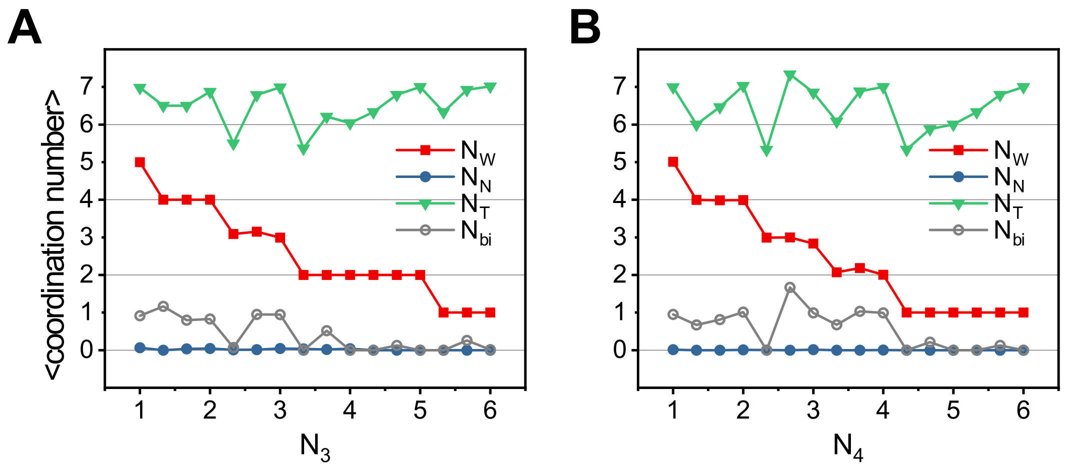

Fig E: Number of coordinated water molecules (N_W), non-native ligands (N_N) as well as the total coordination number (N_T) as functions of N_3 and N_4 for EF_3 (A) and EF_4 (B). Although only one carboxyl oxygen atom is coordinated to the Ca^{2+} at the native coordination structure, the second carboxyl oxygen atom may also bind to the Ca^{2+} in the intermediate state of the coordination. The average number of such non-native bidentate ligand was also shown in panels A and B (N_{bi}, gray).

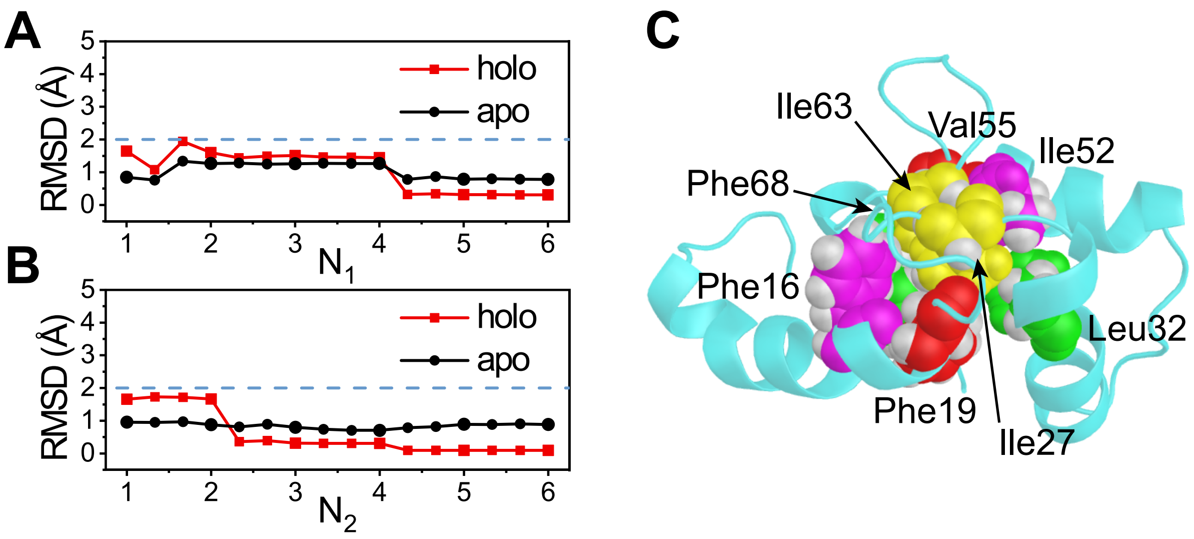

Fig F: Allosteric conformational changes in the hydrophobic cluster of nCaM. (A, B) The RMSD of the hydrophobic residues at conserved positions for the two EF-hand motifs as a function of N_1 (A) and N_2 (B). The relevant residues in the hydrophobic core are Phe16, Phe19, Ile27, Leu32 for EF_1, and Ile52, Val55, Ile63, Phe68 for EF_2. (C) The hydrophobic cluster in the nCaM shows that it is densely packed.

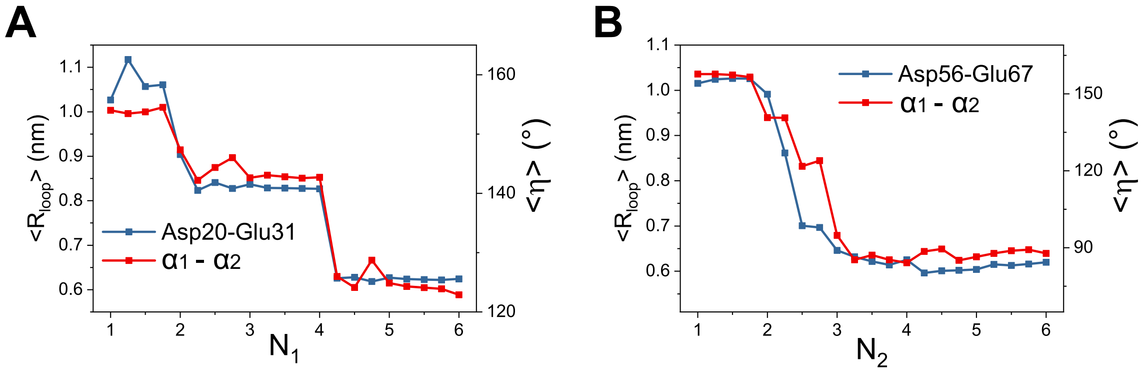

Fig G: Ca^{2+} binding induced rotation of EF-hand helices, characterized using inter-helical angles. (A) Inter-helical angle between the helices in EF_1 as a function of N_1. (B) Inter-helical angle between the helices in EF_2 as a function of N_2. The definition of inter-helical angles are explained in Fig 5C in the main text. The end-end distance of EF-loop is also represented, as the same in Fig 5A in the main text, to show synergistic conformational changes.

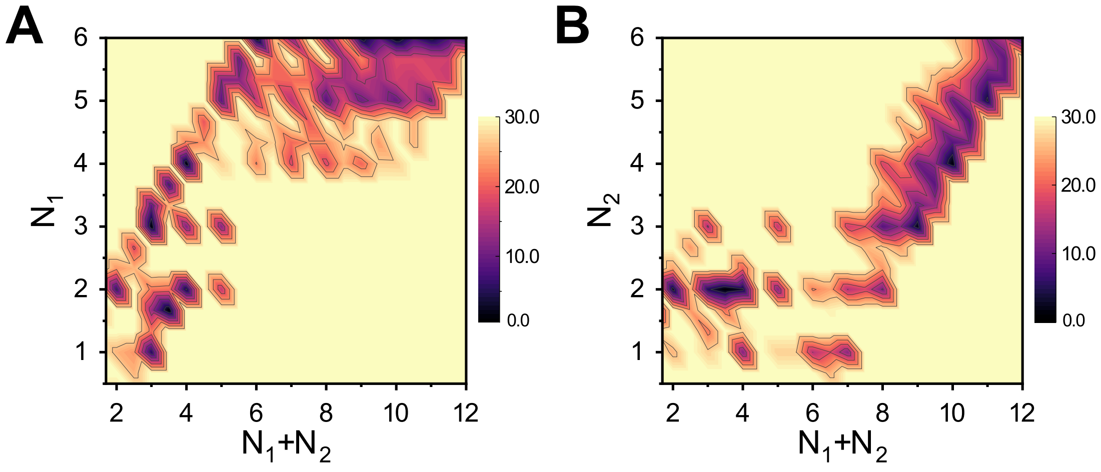

Fig H: Two-dimensional free energy profiles illustrating the coupling of Ca^{2+} coordinations between EF1 and EF2. (A, B) Free energy profiles projected onto the collective variables ($N_1 + N_2$, N_1) (A) and ($N_1 + N_2$, S_2) (B), respectively. The free energy scales are in kJ/mol.

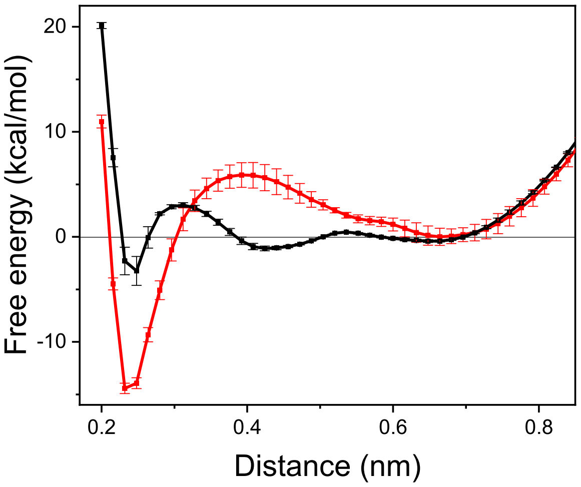

Fig I: Potential of mean force for the binding/unbinding process of Glu31 to Ca^{2+} as a function of the minimum distance between side-chain oxygen atoms of Glu31 and Ca^{2+} using modified parameter set of LJ potential function described by Kahlen and coworkers[9]. The black line illustrates the results with the water molecules being realistically treated. The red line shows the results with electrostatic interactions involving the bridging water molecules being turned off. The error bars represent the difference between the results from two groups of simulations, each group containing 10 independent metadynamics simulations. Compared to Fig 6A shown in the main text, the native coordination states corresponding to the free energy minimum around the distance of ∼0.24 nm are less stable. However, after switching off the electrostatic interactions between the bridging water and the Glu31 side-chain oxygen atoms in the control simulation, the free energy barrier for the ligand exchange becomes much higher, suggesting that the bridging water tends to reduce the ruggedness of the ligand exchange free energy landscape, which is consistent with the results shown in Fig 6A based on intact OPLS-AA force field.

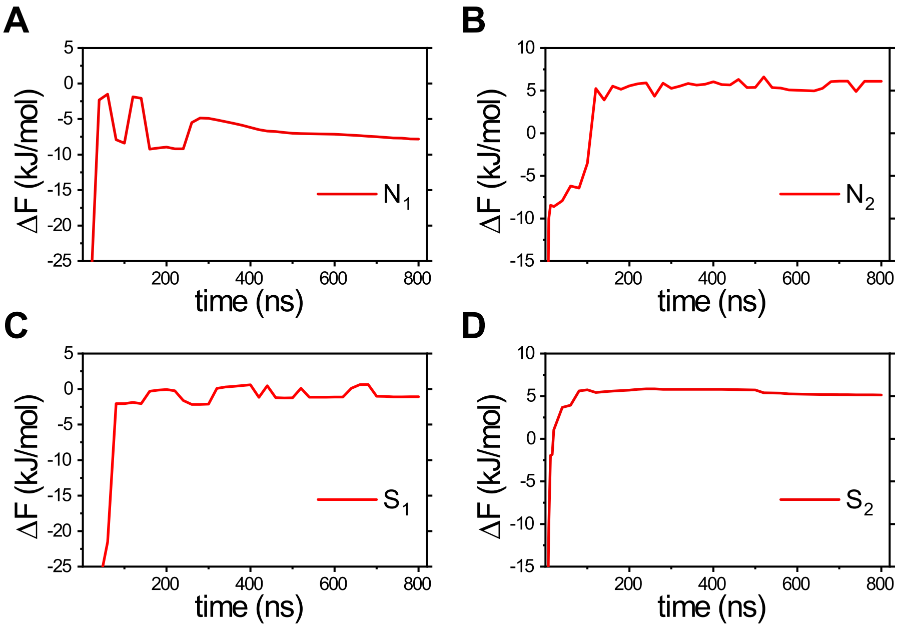

Fig J: The free energy difference between local minima in a one dimensional FES corresponding to a specific CV as a function of the simulation time. (A) and (B) Free energy differences between the regions at $N_\alpha = 6$ and $N_\alpha = 2$ for EF_1 and EF_2 ($\Delta F = F(N_\alpha = 6) - F(N_\alpha = 2)$, $\alpha = 1, 2$). (C) and (D) Free energy differences between the open conformation and the closed conformation for the two EF-hands ($\Delta F = F(S_\alpha < 1.2) - F(S_\alpha > 1.8)$, $\alpha = 1, 2$). In (A-D) the ΔFs reach steady values after ∼300 ns of simulations, which indicate the convergence of metadynamics simulations.

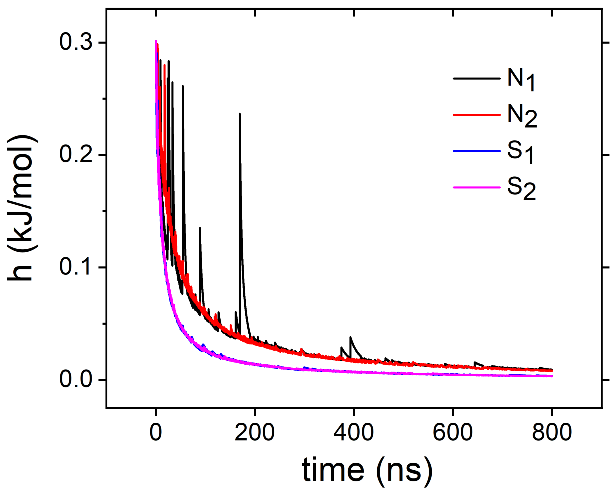

Fig K: Height of the biasing Gaussian potentials (defined in Supplementary Equation (S2)) in the metadynamics as functions of simulation time. In our well-tempered metadynamics simulations, the initial value of the Gaussian height was set to 0.3kJ/mol, while the bias factor $\eta = 200$ was used to control the rate of potential decrease. After 200 ns, all the biasing Gaussians are smaller than 0.05kJ/mol, which further decrease to values less than 0.03kJ/mol after 300ns. These correspond to only about 1/10 of the initial values.

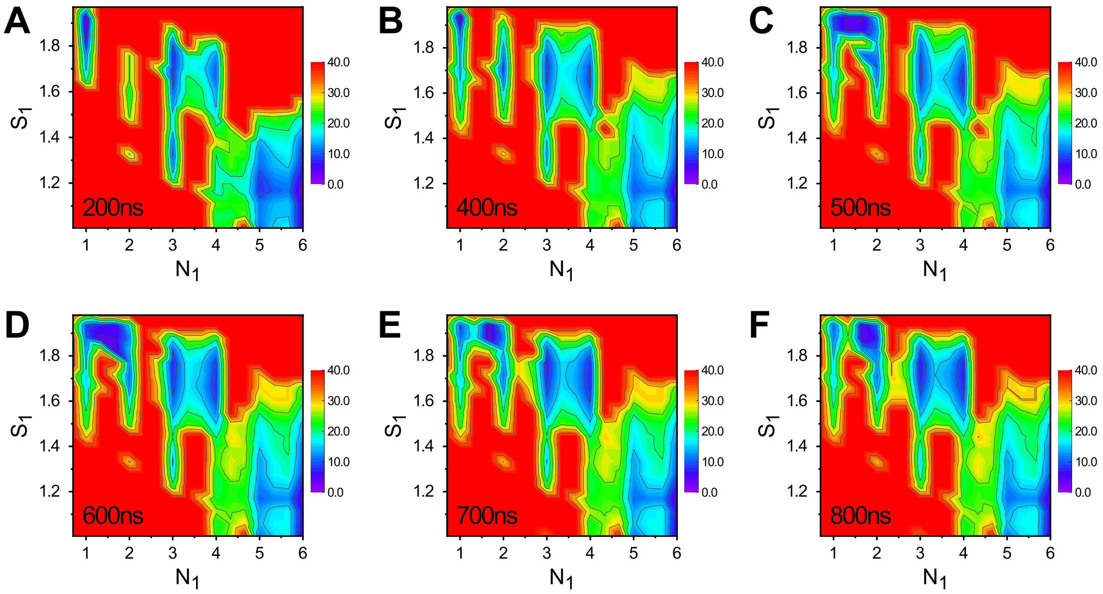

Fig L: Time evolution of the two-dimensional N_1-S_1 free energy surface sampled by well-tempered bias-exchange metadynamics. Note that after 400 ns the two dimensional surfaces are stationary, indicating the convergence of the simulation results.
